# Increased deposition of C3b on red cells with low CR1 and CD55 in a malaria-endemic region of western Kenya: Implications for the development of severe anemia

**DOI:** 10.1186/1475-2875-9-S2-P28

**Published:** 2010-10-20

**Authors:** Collins O Odhiambo, Walter Otieno, Christine Adhiambo, Michael M Odera, José A Stoute

**Affiliations:** 1 The US Army Medical Research Unit and the Kenya Medical Research Institute, Nairobi, Kenya; 2 Department of Medicine, the Uniformed Services University of the Health Sciences, Bethesda, Maryland, USA; 3 Division of Malaria Vaccine Development, Department of Cellular Injury, The Walter Reed Army Institute of Research, Robert Grant Avenue, Silver Spring, MD 2091 0, USA; 4 Pennsylvania State College of Medicine, 500 University Drive, MC: H036, Rm C6833, Hershey, PA 17033, USA

## Background

Severe anemia due to *Plasmodium falciparum* malaria is a major cause of mortality among young children in western Kenya. The factors that lead to the age-specific incidence of this anemia are unknown. Previous studies have shown an age-related expression of red cell complement regulatory proteins, which protect erythrocytes from autologous complement attack and destruction. Our primary objective was to determine whether in a malaria-endemic area red cells with low levels of complement regulatory proteins are at increased risk for complement (C3b) deposition *in vivo*. Secondarily, we studied the relationship between red cell complement regulatory protein levels and hemoglobin levels.

## Methods

Three hundred and forty-two life-long residents of a malaria-holoendemic region of western Kenya were enrolled in a cross-sectional study and stratified by age. We measured red cell C3b, CR1, CD55, and immune complex binding capacity by flow cytometry. Individuals who were positive for malaria were treated and blood was collected when they were free of parasitemia. Analysis of variance was used to identify independent variables associated with the %C3bpositive red cells and the hemoglobin level.

## Results

Individuals between the ages of 6 and 36 months had the lowest red cell CR1, highest %C3b-positive red cells and highest parasite density. Malaria prevalence also reached its peak within this age group. Among children ≤ 24 months of age the %C3b-positive red cells was usually higher in individuals who were treated for malaria than in uninfected individuals with similarly low red cell CR1 and CD55. The variables that most strongly influenced the %C3b-positive red cells were age, malaria status, and red cell CD55 level. Although it did not reach statistical significance, red cell CR1 was more important than red cell CD55 among individuals treated for malaria. The variables that most strongly influenced the hemoglobin level were age, the %C3b-positive red cells, red cell CR1, and red cell CD55.

## Conclusion

Increasing malaria prevalence among children >6 to ≤36 months of age in western Kenya, together with low red cell CR1 and CD55 levels, results in increased C3b deposition on red cells and low hemoglobin. The strong contribution of age to C3b deposition suggests that there are still additional unidentified age-related factors that increase the susceptibility of red cells to C3b deposition and destruction.

